# Editorial: 17th Spanish Society for Developmental Biology Meeting: New Trends in Developmental Biology

**DOI:** 10.3389/fcell.2022.897989

**Published:** 2022-04-20

**Authors:** Sofia J. Araújo, Rosa Barrio

**Affiliations:** ^1^ Department of Genetics, Microbiology and Statistics, School of Biology, University of Barcelona, Barcelona, Spain; ^2^ Institute of Biomedicine University of Barcelona (IBUB), Barcelona, Spain; ^3^ Center for Cooperative Research in Biosciences (CIC bioGUNE), Basque Research and Technology Alliance (BRTA), Bizkaia Technology Park, Derio, Spain

**Keywords:** Developmental Biology, Conference, Spanish Society for Developmental Biology, Trends in Development, Scientific Society, Virtual Meeting

The Spanish Society for Developmental Biology organized its 17th meeting in November 2020. The meeting, organized by CIC bioGUNE, the University of the Basque Country and the University of Cantabria, gathered about 280 registrants and received 132 scientific abstracts. Participants ranged from undergraduate to senior researchers, with a broad participation of Ph.D. students. The meeting was organized in 8 sessions: Growth and Scaling, Self-organization, Neurodevelopment, Genomes, Cell Biology, Development and Disease, Evo-Devo and Regeneration (Araújo et al., 2021). These sessions focused on the new tendencies in Developmental Biology research and, based on the science presented there, we organized this special issue on *The 17*th *Edition of the Spanish Society for Developmental Biology Meeting: New Trends in Developmental Biology.* This collection of articles gathers several scientific contributions in this area, featuring collaborative and interdisciplinary approaches among developmental biologists.

With the focus on organogenesis and gene regulation, our selected content embraces novel discoveries on muscle development, regeneration and the transcriptional control and role of miRNAs in development, while highlighting advances in organogenesis and gonadal development.

Original research articles include works on ear morphogenesis, where Durán-Alonso et al. show that Meis2 is essential for inner ear formation, providing an entry point to the network underlying proper cochlear coiling. Muscle morphogenesis is approached in a report by Pérez-Moreno et al., reporting that the interaction between laminins and the proteoglycan Kon-tiki/Perdido controls both myotube migration and attachment during *Drosophila* myogenesis. Garcia-Padilla et al., study the role of Bmp- and Fgf signaling in the modulation of mouse proepicardium cell fate and propose that species-specific differences regulate proepicardial/septum transversum cardiomyogenic lineage commitment. Muscle satellite cell origin and heterogeneity in the context of muscle regeneration is reviewed by Rodriguez-Outeiriño et al., who summarize works supporting the different developmental origins of satellite cells.

Planarian regeneration is tackled by Coronel-Córdoba et al., who study the involvement of FoxK1 transcription factor in ectodermal tissues and report on its involvement neural and epidermal tissue regeneration.

Organogenesis is the subject of more research reported in this issue. Camacho-Macorra et al. analyze the involvement of the Hedgehog pathway in craniofacial development, by studying Mosmo, a modulator of the pathway. Inactivation of the two zebrafish Mosmo paralogs induces craniofacial skeleton defects, suggesting that Mosmo is a candidate to explain uncharacterized forms of human congenital craniofacial malformations. Appendage development is the subject of study of Ruiz-Losada et al., who report on a redundant role of forkhead transcription factors Fd4 and Fd5 during *Drosophila* leg development. The involvement of miRNAs during early cardiac development is studied by Garcia-Padilla et al., who analyze the expression profiles of different miRNAs and demonstrate their modulation of different Hox clusters. They propose that miRNAs are involved in Hox gene modulation during differentiation and compartmentalization of the posterior structures of the developing venous pole of the heart.

Developmental gene regulation is explored by Giordano et al., who report on the relationship between two key transcription factors, CBX4 and SALL1, showing they interact in the nucleoplasm and uncovering a new way of SALL1-mediated regulation of Polycomb bodies through modulation of CBX4 stability. They present a novel mechanism of regulation of a developmental factor, which has consequences for the regulation of its target genes.

Estermann et al. build the first comparative molecular characterization of gonadal supporting cell differentiation in birds, which reveals conservation of PAX2^+^ expression and a mesenchymal origin of gonadal supporting cells, indicating that gonadal morphogenesis is conserved among the major bird clades.

An original research report by Barettino et al. reports on the developmental mechanisms guiding the formation of serotonergic sub-systems. They unveil a developmental process acting through Erb4 in subsets of serotonergic neurons to orchestrate a long-range circuit involved in the formation of emotional and social memories. Nervous system development in the context of the transcriptional control of axon guidance and the selection of trajectories at midline structures is reviewed by Herrera and Escalante.

Finally, Parra-Peralbo et al., review the current knowledge on the origin and development of the adipose tissue, focusing on this organ in *Drosophila melanogaster* and identifying some gaps for future research.

Together, manuscripts in this Research Topic (https://www.frontiersin.org/research-topics/17566/17th-edition-of-the-spanish-society-for-developmental-biology-meeting-new-trends-in-developmental-bi#articles) provide an overview of the latest research on the new trends in developmental biology, ranging from early embryogenesis to regeneration, through gene regulation, in a variety of systems and organs. The contributions presented here reflect the diversity and richness of the presentations we enjoyed at the 17th edition of the SEBD meeting. We are looking forward to the next edition!
